# Supplementary material for: Knowledge and attitude of populations on blackflies and onchocerciasis and participation in mass drug administration in first-line communities near Erin-Ijesha and Arinta waterfalls, Southwest Nigeria
Source: PLoS Negl Trop Dis. 2025 Aug 14;19(8):e0013411. doi: 10.1371/journal.pntd.0013411 (PMC12367129; doi:10.1371/journal.pntd.0013411)
Supplement: S1 File — (DOCX) [file pntd.0013411.s001.docx]

Questionnaire on Knowledge, Practices, and Participation in Mass Administration of Medicine for Control of Onchocerciasis

This questionnaire is to determine the knowledge, practices and the level of participation of community members during community

distribution of ivermectin.

Section 1: Demographic information of participants

1. Location
2. Name
3. Unique ID no
4. Age range

*Mark only one oval.*


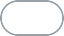
 10 - 20


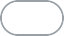
 21 - 40


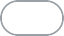
 41 - 60


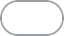
 61 - 80

1. Gender

*Tick all that apply.*


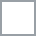
 Male
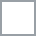
 Female

1. Education

*Mark only one oval.*


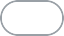
 Primary
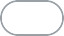
 Secondary
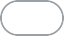
 Tertiary


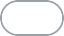
 No formal education

1. Occupation

*Mark only one oval.*


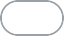
 Student
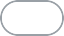
 Farmer
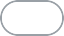
 Trader


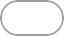
 Civil servant
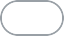
 Retired
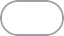
 Unemployed

Section 2: Knowledge and Attitude on Blackfly

1. Have you heard of blackflies?

*Mark only one oval.*


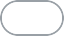
 Yes
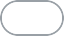
 No

1. Do black flies bite in your community?

*Mark only one oval.*


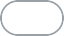
 Yes
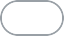
 No

1. Where do these black flies primarily come from?

*Mark only one oval.*


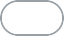
 Trees
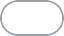
 Rivers
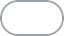
 Others
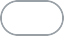
 No idea

1. Which part of the body do black flies mostly bite?

*Mark only one oval.*


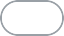
 Head
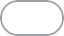
 Hand
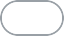
 Legs
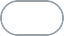
 No idea

1. What are the immediate effects of a black fly bite?

*Mark only one oval.*


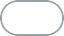
 Itching
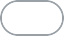
 Swelling
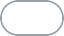
 No idea

1. During which season do black flies bite more frequently?

*Mark only one oval.*


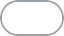
 Dry
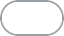
 Rainy

1. What time of day do black flies usually bite?

*Mark only one oval.*


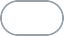
 Day
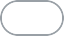
 Night
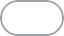
 No idea

Section 3: Knowledge on Onchocerciasis

*Mark only one oval.*


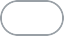
 Option 1

1. Do you know what onchocerciasis is?

*Mark only one oval.*


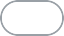
 Yes
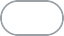
 No

1. What is the local name for onchocerciasis in your community?

*Mark only one oval.*


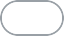
 Sobia
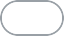
 Naarun


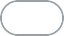
 Arun fopawon
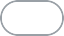
 No idea

1. How is the disease transmitted?

*Mark only one oval.*


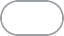
 Mosquito bite
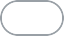
 Blackfly bite
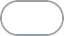
 Witchcraft
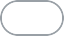
 Hereditary
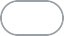
 No idea

1. What are the common symptoms of onchocerciasis?

*Mark only one oval.*


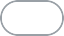
 Nodules
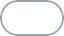
 Leopard skin


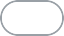
 Ocular dermatitis/Blindness
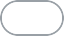
 No idea

1. Are you aware that this disease can cause blindness if not treated?

*Mark only one oval.*


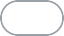
 Yes
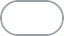
 No

Section 4: Attitude and Level of Participation in CDTi

1. How do you prevent black flies from biting you?

*Mark only one oval.*


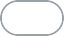
 Wearing socks
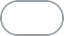
 Clothings
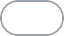
 Leaf extracts
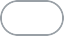
 Ointments
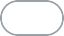
 Nothing

1. Are you aware of any community sensitization efforts regarding onchocerciasis?

*Mark only one oval.*


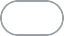
 Yes
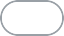
 No

1. Do you know of any local medication or treatment for onchocerciasis?

*Mark only one oval.*


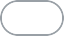
 Yes
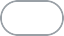
 No

1. If yes, what?
2. Have you heard about ivermectin or Mectizan before?

*Mark only one oval.*


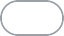
 Yes
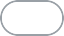
 No

1. **Have you ever received ivermectin before?**

*Mark only one oval.*


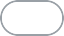
 Yes
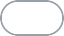
 No

1. If Yes, how many rounds?

*Mark only one oval.*


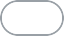
 once
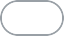
 2


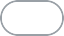
 > 2


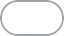
 > 5

1. If you have not received treatment, what is the reason?

*Mark only one oval.*


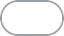
 Not aware of distribution
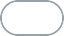
 I just refuse


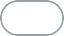
 Not always around
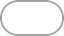
 Other

1. Would you be willing to participate in future CDTi for onchocerciasis?

*Mark only one oval.*


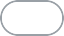
 Yes
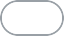
 No

1. If No, why?
2. Do you know how onchocerciasis can be prevented?

*Mark only one oval.*


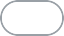
 Yes
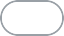
 No

1. (If Yes) Please specify:
2. Are there any measures taken in your community to prevent or control onchocerciasis?

*Mark only one oval.*


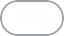
 Yes
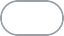
 No

1. (If Yes) Please specify:
2. Have you or anyone in your family received treatment for onchocerciasis?

*Mark only one oval.*


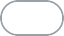
 Yes
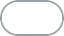
 No

1. (If Yes) How often do they receive treatment?

*Mark only one oval.*


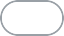
 Every 6 months
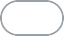
 Annually


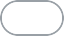
 Others:

1. What treatment or medication do you know of for onchocerciasis?

*Mark only one oval.*


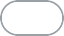
 Ivermectin (mectizan)
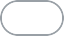
 Herbal medicine


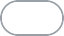
 Others:

1. Do you believe that onchocerciasis can be eliminated in your community?

*Mark only one oval.*


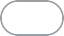
 Yes
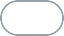
 No
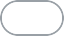
 unsure

1. Do you believe that onchocerciasis can be eliminated in your community?

*Mark only one oval.*

Yes
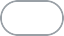
 No
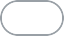
 Unsure


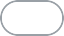


1. Do you believe efforts to sensitize the community about onchocerciasis elimination can be improved?


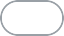
 Yes
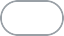
 No
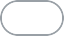
 Unsure

1. (If No) What would help increase awareness?

*Mark only one oval.*


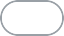
 More health worker outreach


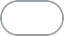
 Radio/TV/social media campaigns Educational sessions in schools

Other:

1. Do you think that everyone in your community understands the importance of taking treatment for onchocerciasis?

*Mark only one oval.*

Yes No Unsure

1. Have you noticed any stigma or discrimination toward those who show symptoms of onchocerciasis in your community?

*Mark only one oval.*

Yes No

1. (If Yes) Please explain
