# Supplementary material for: Knowledge and attitude of populations on blackflies and onchocerciasis and participation in mass drug administration in first-line communities near Erin-Ijesha and Arinta waterfalls, Southwest Nigeria
Source: PLoS Negl Trop Dis. 2025 Aug 14;19(8):e0013411. doi: 10.1371/journal.pntd.0013411 (PMC12367129; doi:10.1371/journal.pntd.0013411)
Supplement: Attachment — Submitted filename: PNTD-D-24-01867_reviewer.pdf [file pntd.0013411.s004.pdf]

PLOS Neglected Tropical Diseases

Knowledge and attitude on blackflies and onchocerciasis and participation in Mass Drug Administration in frontline communities near Erin-Ijesha and Arinta waterfalls, Southwest Nigeria.

--Manuscript Draft--

**Manuscript Number:**	PNTD-D-24-01867
**Full Title:**	Knowledge and attitude on blackflies and onchocerciasis and participation in Mass Drug Administration in frontline communities near Erin-Ijesha and Arinta waterfalls, Southwest Nigeria.
**Short Title:**	Knowledge and practices towards blackflies and onchocerciasis in Southwest Nigeria.
**Article Type:**	Research Article
**Keywords:**	Knowledge and attitude; Blackflies; Onchocerciasis; Mass Drug Adminitration; Southwest Nigeria
**Abstract:**	Background<br><br>Preliminary assessments identified blackfly biting activity at Erin-Ijesha and Arinta waterfalls in Southwest Nigeria, previously unmapped for onchocerciasis transmission. There is a need to assess knowledge of blackflies, onchocerciasis, and participation in treatment programs among residents of first-line communities near the waterfalls to evaluate onchocerciasis transmission risk.<br><br>Methods<br><br>A cross-sectional survey was conducted among 236 residents at Erin-Ijesha and Ipole-Iloro, two front-line communities using structured questionnaires. Data were entered into Microsoft Excel and analyzed using the Statistical Package for the Social Sciences (SPSS) software version 21. Relationships between variables were analyzed using t-test and chi-square, with a 95% confidence interval.<br><br>Results<br><br>Majority (73.3%) in Erin-Ijesha and (83.7%) in Ipole-Iloro reported knowledge of blackflies, with 83.3% and 87.0% aware of onchocerciasis, respectively. The majority of participants had no knowledge of how onchocerciasis is transmitted, with only 8.7% and 10.5% in Erin-Ijesha and Ipole-Iloro, respectively, linking the disease to blackfly bites. Ivermectin uptake was relatively high at 75.7% and 76.7% in Erin-Ijesha and Ipole-Iloro, respectively, but still below the 80% therapeutic coverage recommended by World Health Organization (WHO). Willingness to participate in future treatment programs was lower, at 64.7% and 64.0% in Erin-Ijesha and Ipole-Iloro, respectively. Level of education significantly influenced willingness to participate ($p < 0.05$).<br><br>Conclusion<br><br>The study highlights low level of knowledge of onchocerciasis transmission and the barriers to achieving optimal treatment coverage. Addressing these challenges requires integrating health education into schools, expanding health worker outreach, and enhancing community-level media campaigns.
**Additional Information:**	
**Question**	**Response**
**Government Employee**<br><br>Are you or any of the contributing authors an employee of the United States government?	No - No authors are employees of the U.S. government.

Powered by Editorial Manager® and ProduXion Manager® from Aries Systems Corporation

Manuscripts authored by one or more US Government employees are not copyrighted, but are licensed under a CC0 Public Domain Dedication, which allows unlimited distribution and reuse of the article for any lawful purpose. This is a legal requirement for US Government employees.<br><br>This will be typeset if the manuscript is accepted for publication.	
**Financial Disclosure**<br><br>Enter a financial disclosure statement that describes the sources of funding for the work included in this submission. Review the submission guidelines for detailed requirements. View published research articles from *PLOS Neglected Tropical Diseases* for specific examples.<br><br>This statement is required for submission and **will appear in the published article** if the submission is accepted. Please make sure it is accurate.<br><br>**Funded studies**<br>Enter a statement with the following details:<br>• Initials of the authors who received each award<br>• Grant numbers awarded to each author<br>• The full name of each funder<br>• URL of each funder website<br>• Did the sponsors or funders play any role in the study design, data collection and analysis, decision to publish, or preparation of the manuscript?<br><br>Did you receive funding for this work?	The author(s) received no specific funding for this work.
**Competing Interests**<br><br>On behalf of all authors, disclose any competing interests that could be perceived to bias this work.	The authors declare no competing interests.

This statement will be typeset if the manuscript is accepted for publication.<br><br>Review the instructions link below and PLOS NTDs' competing interests policy to determine what information must be disclosed at submission.	
**Data Availability**<br><br>Provide a **Data Availability Statement** in the box below. This statement should detail where the data used in this submission can be accessed. This statement will be typeset if the manuscript is accepted for publication.<br><br>Before publication, authors are required to make all data underlying their findings fully available, without restriction. Review our PLOS Data Policy page for detailed information on this policy. Instructions for writing your Data Availability statement can be accessed via the Instructions link below.	All data generated and/or analysed during this study will be made available by the corresponding author upon reasonable request

Powered by Editorial Manager® and ProduXion Manager® from Aries Systems Corporation

Manuscript Click here to access/download;Manuscript;Manuscript.docx

Knowledge and attitude on blackflies and onchocerciasis and participation in Mass Drug Administration in frontline communities near Erin-Ijesha and Arinta waterfalls, Southwest Nigeria.

Oluwadamilare Ganiu Dauda[1*], Akinlabi Mohammed Rufai[1], Olabanji Ahmed Surakat[1], Oluwatoyin Adeola Oyeniran[2], Olusegun Quadri Adeshina[1], Abuh Ojomona Oboro[1], Azeezat Ayanbukola Ayannibi[3], Rhidollah Folashade Oyewusi[1], Glory Blessing Jokanola[1], Kamilu Ayo Fasasi[1], Monsuru Adebayo Adeleke[1].

[1]Department of Animal and Environmental Biology, Osun State University, Osogbo, Osun State, Nigeria.

[2]Department of Medical Microbiology and Parasitology, Osun State University Teaching Hospital, Osogbo, Osun State.

[3]Department of Pure and Applied Biology, Ladoke Akintola University of Technology, Ogbomoso, Oyo State, Nigeria.

Abstract

Background: Preliminary assessments identified blackfly biting activity at Erin-Ijesha and Arinta waterfalls in Southwest Nigeria, previously unmapped for onchocerciasis transmission. There is a need to assess knowledge of blackflies, onchocerciasis, and participation in treatment programs among residents of first-line communities near the waterfalls to evaluate onchocerciasis transmission risk.

Methods: A cross-sectional survey was conducted among 236 residents at Erin-Ijesha and Ipole-Iloro, two front-line communities using structured questionnaires. Data were entered into Microsoft Excel and analyzed using the Statistical Package for the Social Sciences (SPSS) software version 21. Relationships between variables were analyzed using t-test and chi-square, with a 95% confidence interval.

Results: Majority (73.3%) in Erin-Ijesha and (83.7%) in Ipole-Iloro reported knowledge of blackflies, with 83.3% and 87.0% aware of onchocerciasis, respectively. The majority of participants had no knowledge of how onchocerciasis is transmitted, with only 8.7% and 10.5% in Erin-Ijesha and Ipole-Iloro, respectively, linking the disease to blackfly bites. Ivermectin uptake was relatively high at 75.7% and 76.7% in Erin-Ijesha and Ipole-Iloro, respectively, but still below the 80% therapeutic coverage recommended by World Health Organization (WHO). Willingness to participate in future treatment programs was lower, at 64.7% and 64.0% in Erin-Ijesha and Ipole-Iloro, respectively. Level of education significantly influenced willingness to participate ($p < 0.05$).

Conclusion: The study highlights low level of knowledge of onchocerciasis transmission and the barriers to achieving optimal treatment coverage. Addressing these challenges requires integrating health education into schools, expanding health worker outreach, and enhancing community-level media campaigns.

Author Summary

Preliminary assessments have identified blackfly biting activity at Erin-Ijesha and Arinta waterfalls in Southwest Nigeria, regions previously unmapped for onchocerciasis transmission. These black flies make use of fast flowing rivers for the development of their

immature stages and the adult fly transmits the parasite *Onchocerca volvulus* that causes severe skin and eye disorder. The disease is particularly prevalent in communities near suitable river systems. Erin-Ijesha and Ipole-Iloro, two front-line communities near the waterfalls, have been involved in mass ivermectin distribution programs for years. However, this study reveals a striking lack of knowledge among residents regarding the bioecology of blackflies, severity of onchocerciasis and the importance of ivermectin uptake. This knowledge gap has contributed to suboptimal treatment coverage, with some individuals refusing to participate in annual mass drug distribution. These findings highlight the urgent need for improved community health education, more health worker outreach, and targeted media campaigns to address misconceptions, improve awareness, and optimize treatment program outcomes.

Introduction

Onchocerciasis is a skin disfiguring and blinding tropical infectious disease that significantly impacts affected communities and caused by the filarial nematode *Onchocerca volvulus* and transmitted by *Simulium* vector [1]. The epidemiology of onchocerciasis is influenced by the abundance, biting behaviour and vectorial capacity of local blackfly populations. The disease is found in isolated regions of Latin America and Yemen but the overwhelming majority of the global burden of onchocerciasis is concentrated in Africa, with more than 99% of cases occurring in 31 countries within sub-Saharan Africa [1]. Nigeria holds the highest global burden of onchocerciasis, accounting for approximately 40% of all cases worldwide [2,3]. The disease manifests through skin lesions accompanied by intense itching, as well as severe eye damage that can lead to

blindness [1,4]. It is actually the second leading cause of preventable blindness in the world behind trachoma [5]

In Africa, onchocerciasis intervention programs shifted their goal from merely managing the disease as a public health issue by achieving a reduction in skin and ocular complications to targeting the elimination of the disease [6]. This change was driven by evidence from several studies demonstrating that *Onchocerca volvulus* elimination is achievable through community-directed ivermectin treatment (CDTI) [7-9]. Based on this evidence, disease control efforts in Nigeria have also primarily focused on mass drug administration (MDA) with ivermectin, a strategy that has been implemented for nearly 20 years, with states progressing at varying stages toward elimination [2]. This approach is crucial as it empowers endemic communities to decide the timing of drug distribution, choose their distributors, determine the distribution method, and take part in supervising mass drug administration [6]. Currently, ivermectin has been administered to over 45 million Nigerians across more than 36,000 communities, achieving over 78% of the ultimate treatment goal [2].

A preliminary survey of rivers in Southwest Nigeria revealed blackfly activity at two major tourist sites, the Erin-Ijesha and Arinta waterfalls in Osun and Ekiti States, respectively, where such biting activity had not been previously reported or documented. Both states were classified as mesoendemic for onchocerciasis based on several surveys using the Rapid Epidemiological Mapping of Onchocerciasis (REMO) mapping conducted in 1995–1996 [2] and have been receiving ivermectin treatment since 2000 [10]. Both states are considered to be on track for disease elimination [2]. However, the Erin-Ijesha and Arinta waterfalls were not previously included in onchocerciasis mapping efforts in both states,

and there is no evidence-based documentation on the infectivity status of blackflies at these sites in order to determine the transmission risk of onchocerciasis in first-line communities nearest to the waterfalls. The absence of data on onchocerciasis prevalence in these two regions, despite the observed blackfly activity, raises concerns about potential transmission dynamics, given the vector's role in the epidemiology of the disease. This study was designed as part of a broader epidemiological survey to evaluate community knowledge, attitudes, and determine level of participation in ivermectin mass drug administration (MDA) in order to assess the onchocerciasis transmission risk in the region. This information will be crucial for the state ministries of health and implementing partners to design effective control strategies aimed at achieving optimal therapeutic coverage in both states.

Materials and Methods

Study area

This study was conducted between October and November 2024 in two frontline communities near the Arinta and Olumirin waterfalls. The first site, Erin Ijesha (N7.569368, E4.891340), located in Oriade Local Government Area, Osun State, is the nearest community to the Erin-Ijesha waterfall. The second site, Ipole Iloro (N7.586372, E4.936383), situated in Ekiti West Local Government Area, Ekiti State, is the frontline community to the Arinta waterfall. These communities have not been previously mapped, and no pre-control data on onchocerciasis prevalence are available. However, blackfly activity observed during a preliminary assessment prompted the selection of these locations for the study (Figure 1).

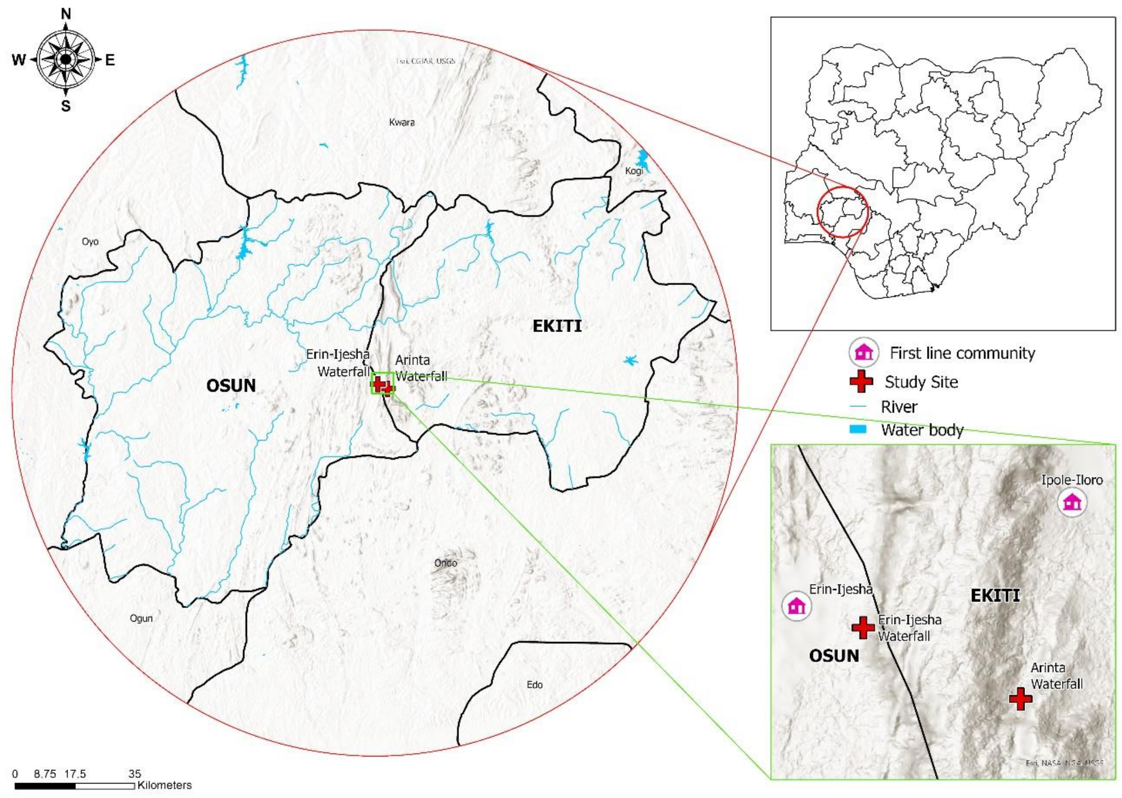

Fig 1. Map showing study locations and the waterfalls

Ethical considerations

The ethical approval for this study was granted by the Ethics Review Committees of the Osun State Ministry of Health (reference number OSHREC/PRS/569T/334) and the Ekiti State Ministry of Health (reference number MOH/EKHREC/EA/P/50). Informed consent was first obtained from community leaders and religious heads of the communities. For participants aged 10 and below 18 years, recruitment was conducted with the written consent of their parents or guardians after explaining the study's objectives in English and in the local language (Yoruba). Written informed consent was also obtained from participants above 18 years. All participants were informed that participation was voluntary and that they could withdraw at any point without any negative consequences

to them. The research adhered to international ethical standards, including the 1964 Helsinki Declaration and its subsequent amendments.

Study design and determination of sample size

This community-based cross-sectional study was carried out in two selected communities between October 2024 and November 2024. Local neglected tropical disease coordinators (LNTDs), facility health workers from the community primary health centers (PHCs), and local mobilizers facilitated community sensitization and mobilization efforts. Participants aged 10 years and above ~~were enrolled in the study~~ and who have been resident in the communities for 5 years and above. In the absence of prior data on community knowledge of blackflies and onchocerciasis, Cochran's formula was used to determine sample size. It was assumed that 50% of the target population (individuals aged 10 years and above) would possess such knowledge. Using a 95% confidence level and a 5% margin of error, the calculated sample size was 385.

Data collection and analysis

Following informed consent, four trained personnel administered the questionnaire with assistance from a local guide who translated the questions into the local language to ensure clarity and comprehension. The questionnaire comprised four sections: (i) socio-demographic information, (ii) knowledge of blackflies, (iii) knowledge of onchocerciasis, and (iv) attitudes and participation in mass ivermectin administration. Data were entered into Microsoft Excel and analyzed using the 2021 version of the Statistical Package for the Social Sciences (SPSS). Descriptive statistics were performed, and relationships

between variables were assessed using t-tests and chi-square tests, with proportions analyzed at a 95% confidence interval.

Results

Socio-demographic characteristics of study participants

A total of 236 respondents from the two communities participated in the study, with 150 individuals from Erin-Ijesha and 86 from Ipole Iloro. Female participants accounted for the majority (78.4%), while males constituted 21.6%. The largest age group represented was 61–80 years (44.1%) while the lowest age group represented were between the ages 10-20 (4.6%). Respondents with no formal education comprised the highest proportion (34.3%), whereas those with tertiary education were the least represented (7.7%) (Table 1).

Table 1. Socio-demographic characteristics of participants.

Variables	No of individuals at Erin-Ijesha (%)	No of individuals at Ipole Iloro (%)	Total number (%)
Numbers	150	86	236
**Age**			
10-20	8 (5.3)	3 (3.5)	11 (4.6)
21-40	32 (21.3)	4 (4.7)	36 (15.3)
41-60	58 (38.7)	27 (31.4)	85 (36.0)
61-80	52 (34.7)	52 (60.4)	104 (44.1)

**Sex**			
Male	31 (20.7)	20 (23.3)	51 (21.6)
Female	119 (79.3)	66 (76.7)	185 (78.4)
**Education**			
Primary	34 (24.7)	35 (40.7)	69 (29.2)
Secondary	55 (36.7)	13 (15.1)	68 (28.8)
Tertiary	12 (8.0)	6 (7.0)	18 (7.7)
No formal education	49 (32.6)	32 (37.2)	81 (34.3)
**Occupation**			
Student	10 (6.7)	3 (3.5)	13 (5.5)
Farmer	33 (22.0)	41 (47.7)	74 (31.4)
Trader	77 (51.3)	12 (14.0)	89 (37.7)
Civil Servant	6 (4.0)	6 (6.9)	12 (5.1)
Retired	15 (10.0)	10 (11.6)	25 (10.6)
Unemployed	9 (6.0)	14 (16.3)	23 (9.7)

Knowledge on blackfly and bioecology of blackfly

Knowledge about blackflies varied among participants in the two communities. In Erin-Ijesha, 110 (73.3%) of respondents reported having heard of blackflies, while 40 (26.7%) had no knowledge. Additionally, 105 (70.0%) identified blackflies as a biting nuisance in the community, whereas 45 (30.0%) were unaware of this activity. In Ipole-Iloro, 72 (83.7%) of participants were familiar with blackflies, compared to 14 (16.3%) who have no knowledge. Furthermore, 70 (81.4%) confirmed the biting activity of blackflies in the

community, while 16 (18.6%) lacked awareness. When prompted about the origin of blackflies, 81 (54.0%) of respondents in Erin-Ijesha attributed their presence to trees, 56 (37.3%) had no idea, and only 3 (2.0%) correctly identified rivers as their source. Similarly, in Ipole-Iloro, 63 (73.3%) believed blackflies originated from trees, 21 (24.4%) were unsure, and only 2 (2.3%) recognized rivers as their breeding sites (Table 2).

Table 2. Knowledge on blackflies and bioecology.

Questions	Response	Erin-Ijesha (%)	Ipole-Iloro (%)
Have you heard of blackflies	Yes	110 (73.3)	72 (83.7)
	No	40 (26.7)	14 (16.3)
Do black flies bite in your community?	Yes	105 (70.0)	70 (81.4)
	No	45 (30.0)	30 (34.9)
Where do these blackflies primarily come from?	Trees	81 (54.0)	63 (73.3)
	Rivers	3 (2.0)	2 (2.3)
	Others	10 (6.7)	0 (0)
	No idea	56 (37.3)	21 (24.4)

Which part of the body do black flies mostly bite?	Head	1 (0.7)	1 (1.2)
	Hand	16 (10.7)	6 (6.9)
	Legs	86 (57.3)	62 (72.1)
	No idea	47 (31.3)	17 (19.8)
What are the immediate effects of a black fly bite?	Itching	97 (64.7)	66 (76.7)
	Swelling	5 (3.3)	0 (0.0)
	No idea	48 (32.0)	20 (23.3)
During which season do black flies bite more frequently?	Dry	69 (46.0)	46 (53.5)
	Rainy	81 (54.0)	40 (46.5)
What time of day do black flies usually bite?	Day	75 (50.0)	63 (73.3)

	Night	27 (18.0)	5 (5.8)
	No idea	48 (32.0)	18 (20.9)
How do you prevent black flies from biting you?	Wearing socks	11 (7.3)	7 (8.1)
	Clothings	68 (45.3)	37 (43.0)
	Leaf extract	1 (0.7)	1 (1.2)
	Ointments	8 (5.3)	6 (7.0)
	Nothing	62 (41.3)	35 (40.7)

Knowledge on onchocerciasis

At Erin-Ijesha, 125 (83.3%) of respondents demonstrated knowledge and awareness of onchocerciasis, with 88.0% of the respondents accurately identifying the local name for the disease in their community. Similarly, at Ipole-Iloro, 74 (86.0%) of participants were familiar with the disease, and 75 (87.2%) of community members correctly recognized its local name. Despite high awareness levels of the disease, knowledge about the mode of transmission of onchocerciasis was notably poor in both communities. At Erin-Ijesha, 76.7% of respondents have no idea of the disease's transmission route, while at Ipole-Iloro, 82.5% of the respondents also had no idea. Only 8.7% and 10.5% of respondents in Erin-Ijesha and Ipole-Iloro, respectively, correctly identified the blackfly as the vector responsible for the transmission. Misconceptions about the disease were prevalent, and knowledge of its common symptoms was limited. In Erin-Ijesha, 40.0% of respondents

reported having no knowledge of the symptoms, while in Ipole-Iloro, this lack of knowledge was observed in 41.9% of the respondents (Table 3).

Table 3. Knowledge of participants on onchocerciasis

Questions	Response	Erin-Ijesha (%)	Ipole-Iloro (%)
Do you know what onchocerciasis is?	Yes	125 (83.3)	74 (86.0)
	No	25 (16.7)	12 (14.0)
What is the local name for onchocerciasis in your community?	Sobia	0 (0.0)	0 (0.0)
	Naarun	133 (88.7)	75 (87.2)
	Arun fopawon	0 (0.0)	0 (0.0)
	No idea	17 (11.3)	11 (12.8)
How is the disease transmitted?	Mosquito bite	1 (0.7)	3 (3.5)
	Blackfly bite	13 (8.7)	9 (10.5)
	Witchcraft	0 (0.0)	0 (0.0)
	Hereditary	21 (14.0)	3 (3.5)

	No idea	115 (76.7)	71 (82.5)
What are the common symptoms of onchocerciasis?	Nodules	12 (8.0)	9 (10.5)
	Leopard skin	70 (46.7)	18 (20.9)
	Ocular dermatitis/Blindness	8 (5.3)	23 (26.7)
	No idea	60 (40.0)	36 (41.9)
Are you aware that this disease can lead to blindness if not treated?	Yes	69 (46.0)	40 (46.5)
	No	81 (54.0)	46 (53.5)

Attitude and level of participation in mass administration of medicine (MAM)

Awareness of ivermectin was high in both communities, with 127 (84.7%) in Erin-Ijesha and 73 (84.9%) in Ipole-Iloro having heard about the drug. Among those aware, 113 (75.3%) in Erin-Ijesha and 66 (77.6%) in Ipole-Iloro reported receiving at least one dose of ivermectin. When asked about the number of ivermectin doses received, 23 (15.3%) of respondents in Erin-Ijesha reported receiving one dose, 31 (20.7%) two doses, 30 (20.0%) more than two doses, and 29 (19.3%) more than five doses. At Ipole-Iloro, 16 (24.2%) had received one dose, 11 (16.7%) two doses, 22 (33.3%) more than two doses, and 17 (25.8%) more than five doses. Among those 37 (24.7%) and 20 (23.3%)

respondents in Erin-Ijesha and Ipole-Iloro respectively who had not received ivermectin, 11 (29.7%) in Erin-Ijesha and 8 (40.0%) in Ipole-Iloro cited refusal as the reason, while 10 (27.0%) and 9 (45.0%), respectively, reported unawareness of the drug distribution. Regarding willingness to participate in future ivermectin campaigns, 97 (64.7%) of respondents in Erin-Ijesha and 55 (64.0%) in Ipole-Iloro expressed willingness, whereas 53 (35.3%) and 31 (36.0%) respectively, declined.

In Erin-Ijesha, 74 (49.3%) respondents reported being aware of community sensitization efforts on onchocerciasis, while 76 (50.7%) indicated a lack of awareness. Similarly, at Ipole-Iloro, 47 (54.7%) were aware of such efforts, while 39 (45.3%) were not. When asked if sensitization efforts on onchocerciasis elimination could be improved, 116 (77.3%) in Erin-Ijesha and 77 (89.5%) in Ipole-Iloro responded affirmatively. Regarding suggestions for improvement in sensitization efforts in Erin-Ijesha, 44 (29.3%) recommended increased health worker outreach, while 12 (8.0%) suggested leveraging radio, TV, and social media campaigns. In Ipole-Iloro, 13 (15.1%) similarly advocated for more health worker outreach, and 13 (15.1%) also supported the use of radio, TV, and social media for sensitization campaigns. In Erin-Ijesha, education level was found to significantly influenced willingness to participate in future mass administration of ivermectin ($p = 0.00$). Additionally, significant association was also observed between educational level and willingness to participate in future mass administration of ivermectin in Ipole-Iloro ($p = 0.04$). (Table 4).

Table 4. Attitude and level of participation in mass administration of medicine (MAM)

Questions	Response	Erin-Ijesha (%)	Ipole-Iloro (%)
Have you heard about ivermectin or Mectizan before?	Yes	127 (84.7)	73 (84.9)
	No	23 (15.3)	13 (15.1)
Have you ever received ivermectin before?	Yes	113 (75.3)	66 (76.7)
	No	37 (24.7)	20 (23.3)
If Yes, how many rounds?	once	23 (15.3)	16 (24.2)
	Twice	31 (20.7)	11 (16.7)
	> 2 times	30 (20.0)	22 (33.3)
	> 5 times	29 (19.3)	17 (25.8)
If you have not received treatment, what is the reason?	Not aware of distribution	10 (27.0)	9 (45.0)
	I just refuse	11 (29.7)	8 (40.0)
	Not always around	4 (10.8)	2 (10.0)
	Others	12 (32.4)	1 (5.0)
Do you know of any local medication or	Yes	36 (24.0)	14 (16.3)

treatment for onchocerciasis?			
	No	114 (76.0)	72 (83.7)
Are you aware of any community sensitization efforts regarding onchocerciasis?	Yes	74 (49.3)	47 (54.7)
	No	76 (50.7)	39 (45.3)
Do you believe efforts to sensitize the community about onchocerciasis elimination can be improved?	Yes	116 (77.3)	77 (89.5)
	No	4 (2.7)	1 (1.2)
	Unsure	30 (20.0)	8 (9.3)
What would help increase awareness and uptake of ivermectin?	More health worker outreach	44 (29.3)	13 (15.1)

	Radio/TV/social media campaigns	12 (8.0)	13 (15.1)
	Educational sessions in schools	10 (6.7)	4 (4.7)
	Others	24 (16.0)	7 (8.1)
	No response	60 (40.0)	49 (56.9)
Have you noticed any stigma or discrimination toward those who show symptoms of onchocerciasis in your community?	Yes	6 (4.0)	0 (0.0)
	No	144 (96.0)	86 (100)
Would you be willing to participate in future CDTi for onchocerciasis?	Yes	97 (64.7)	55 (64.0)
	No	53 (35.3)	31 (36.0)

Discussion

This study was carried out in the study communities based on outcome of a preliminary breeding site assessment of rivers in Southwest Nigeria that revealed the biting activity

of black flies at the Erin-Ijesha and Arinta waterfall. The high biting density observed at the waterfalls necessitated the need to conduct a knowledge, attitude and practice of community members with regards to blackfly and its ecology, onchocerciasis and level of participation in treatment programs in first-line communities to the waterfalls to determine the transmission risk of onchocerciasis in the communities.

Majority of participants in both communities demonstrated awareness of blackflies with most acknowledging the biting nuisance they cause. However, despite this impressive knowledge, the majority lacked an understanding of the bioecology of blackflies. Misconceptions were widespread, with only 2.0% of respondents in Erin-Ijesha and 2.3% in Ipole-Iloro correctly identifying that blackflies breed in fast-flowing rivers, while the majority believed that the flies originate from trees. This misunderstanding aligns with previously documented misconceptions [11-13]. The lack of knowledge about blackfly bioecology among residents in transmission-risk communities may stem from onchocerciasis control efforts historically focusing on parasite through mass drug administration, with little to non-existent vector control implementation in communities. This poor knowledge could inadvertently increase exposure to blackfly bites, thereby increasing the transmission risk of onchocerciasis in these communities. Occupation and their level of education were not found to have any significant influence on knowledge on bioecology of blackflies in the two communities ($p>0.05$)

There were variations in participants' knowledge of seasonal black fly biting activity across the study communities. In Erin, 54% of participants reported that black flies bite more during the wet season while in Ipole-Iloro, 53.5% believed biting intensity is higher in the dry season. This contrasts with other studies, such as [12] where most participants

associated higher biting frequency with the wet season. These variations may stem from regional differences in vector dynamics. Some entomological studies have reported greater black fly densities during dry season than wet season [14,15], attributed to ecological and climatic factors specific to different *Simulium* species in different ecological zones. Majority of the respondents identified itching as an immediate effect of blackfly bites. This after-effect occurs when the flies inject saliva containing irritant compounds from the epidermal to the dermal layer of the skin, triggering histamine receptors and causing itching along with other skin responses [13,16]. In response to the painful bites of blackflies, most respondents reported using protective measures such as wearing clothing, socks, and applying ointments. These practices align with findings from previous studies conducted across Africa [4,12,17]. Adeleke et al. [11] also documented the use of chemical mixtures, including diesel oil, palm oil, kerosene, and leaf extracts, as repellents. While effective, such substances pose health risks when applied directly to the skin. While many take precautionary measures against the flies, a significant proportion of respondents, 41.3% in Erin-Ijesha and 40.7% in Ipole-Iloro reported taking no measures to prevent bites. This lack of protective behaviour leaves a substantial number of the population exposed to bites, thereby increasing the risk of onchocerciasis transmission within these communities.

An interesting finding is that the majority of participants demonstrated awareness of onchocerciasis and correctly identified its local name, "naarun." Similar patterns of knowledge have been observed in other studies, where most respondents recognized the disease and provided its local name [18,19]. This high level of awareness is likely attributable to the mass drug administration (MDA) campaigns with ivermectin, which

have been ongoing in these communities for over two decades. However, despite this encouraging disease awareness, most participants, 76.7% in Erin-Ijesha and 82.5% in Ipole-Iloro were unaware of how onchocerciasis is transmitted. Only 8.7% and 10.5% in Erin-Ijesha and Ipole-Iloro, respectively, associated the disease with blackfly bites. This limited knowledge of the vector is a reflection of the insufficient community sensitization and education efforts. This low knowledge can contribute to increased transmission risk as individuals are less likely to take preventive measures when unaware of the risks posed by blackfly exposure. Additionally, many participants reported no knowledge of the disease's symptoms or its potential to cause blindness. Comparable findings have been documented in other southern regions of Nigeria, where study participants failed to link the ocular symptoms of onchocerciasis to blackfly bites [18,20]. These findings underscore a significant shortfall in community health education and sensitization, despite years of sustained drug distribution efforts.

A notable finding from this study is the therapeutic coverage observed in both communities. Majority of participants were aware of ivermectin with 75.3% in Erin-Ijesha and 76.7% in Ipole-Iloro reporting they had received at least one round of treatment. This treatment coverage falls below the World Health Organization's (WHO) recommended 80% therapeutic target for onchocerciasis control in endemic areas [21]. Furthermore, 19.3% in Erin-Ijesha and 25.8% in Ipole-Iloro indicated they had undergone more than five rounds of annual ivermectin administration. Among those who had not participated in the treatment programs, 29.7% in Erin and 40.0% in Ipole were persistent drug refusers, citing no specific reasons for rejecting the medication. Others were either unaware of the distribution efforts or unavailable during the campaigns. These findings suggest that

community drug distributors rarely follow up with individuals who miss treatment, limiting both treatment uptake and therapeutic coverage. Similar observations were reported by [19] where individuals excluded due to illness or pregnancy were not revisited, hindering efforts to achieve inclusive coverage. These untreated individuals, who remain as exposed to blackfly bites as those on ivermectin, may act as reservoir hosts and a source of re-infection of onchocerciasis within these communities [22]. The limited understanding of the disease's symptoms contributes to inappropriate alternative treatment-seeking behaviors [23]. Some of the participants claimed to take local herbs for the treatment of onchocerciasis. Conversely, improved awareness is associated with better prevention measures, adherence to treatment, and reduced economic burden [19]. There was no significant relationship between knowledge of onchocerciasis and ivermectin uptake in either community ($p > 0.05$).

The lack of adequate sensitization became evident when participants were asked about their willingness to engage in future mass drug administration programs. Only 64% in Erin-Ijesha and 64.7% in Ipole-Iloro expressed a willingness to participate. However, education was found to significantly influenced this decision in both communities, with higher levels of education correlating with greater acceptance of ivermectin treatment ($p < 0.05$). This underscores the importance of education in improving treatment acceptance, suggesting that more health education in schools, targeted health workers outreach and media sensitization could significantly improve acceptability and uptake of ivermectin in endemic communities.

Conclusion

The study has identified low knowledge of onchocerciasis and key barriers to achieving optimum treatment coverage, including issues of non-compliance, insufficient sensitization, and inadequate health education about onchocerciasis. Addressing these challenges would require integrating health education into school curricula, organizing more health worker outreach, and increasing community-level media campaigns to raise awareness about the disease and its transmission.

Acknowledgements

The authors wish to acknowledge the support of the community leaders of Erin-ijesha and Ipole-Iloro community for their support. We would also like to appreciate the state neglected tropical diseases (NTD) coordinators, local NTD coordinators and local mobilizers for their support towards ensuring the success of this study.

Author contributions

Conceptualization: Monsuru Adebayo Adeleke, Akinlabi Mohammed Rufai, Oluwadamilare Ganiu Dauda

Investigation: Oluwadamilare Ganiu Dauda, Olusegun Quadri Adeshina, Abuh Ojomona Oboro, Azeezat Ayanbukola Ayannibi, Rhidollah Folashade Oyewusi, Glory Blessing Jokanola

Methodology: Oluwadamilare Ganiu Dauda, Olabanji Ahmed Surakat

Formal analysis: Oluwadamilare Ganiu Dauda, Oluwatoyin Adeola Oyeniran

Supervision: Monsuru Adebayo Adeleke, Akinlabi Mohammed Rufai, Kamilu Ayo Fasasi

Writing – original draft: Oluwadamilare Ganiu Dauda, Monsuru Adebayo Adeleke, Akinlabi Mohammed Rufai, Olabanji Ahmed Surakat.

Writing – review & editing: Oluwadamilare Ganiu Dauda, Monsuru Adebayo Adeleke, Kamilu Ayo Fasasi, Olabanji Ahmed Surakat

Funding: The authors received no specific funding for this work.

Data availability All data generated and/or analysed during this study will be made available by the corresponding author upon reasonable request.

Declarations

The ethical approval for this study was granted by the Ethics Review Committees of the Osun State Ministry of Health (reference number OSHREC/PRS/569T/334) and the Ekiti State Ministry of Health (reference number MOH/EKHREC/EA/P/50). Informed consent was first obtained from community leaders and religious heads of the communities. For participants aged 10 and below18 years, recruitment was conducted with the written consent of their parents or guardians after explaining the study's objectives in English and in the local language (Yoruba). Written informed consent was also obtained from participants above 18 years. All participants were informed that participation was voluntary and that they could withdraw at any point without any negative consequences to them. The research adhered to international ethical standards, including the 1964 Helsinki Declaration and its subsequent amendments.

Consent for publication

Not applicable.

Competing interests

The authors declare no competing interests.

References

1. WHO. Onchocerciasis (river blindness). 2022. Available from: https://www.who.int/news-room/fact-sheets/detail/onchocerciasis
2. FMoH. Nigeria Onchocerciasis Elimination Plan. 2017. Available from: https://www.cartercenter.org/resources/pdfs/news/health_publications/river_blindness/nigeria-onchocerciasis-elimination-plan-feb2017.pdf
3. Nwoke BEB, Akpan NM, Cama V, Ekpo UF, Idyorough AE, Mafe MA, et al. Onchocerciasis in Nigeria 1: History of control efforts. Nigerian Journal of Parasitology; 2023.
4. Weldegebreal F, Medhin G, Weldegebriel Z, Legesse M. Assessment of community's knowledge, attitude and practice about onchocerciasis and community directed treatment with Ivermectin in Quara District, north western Ethiopia. Parasit Vectors. 2014;7(1):98.
5. Frallonardo L, Di Gennaro F, Panico GG, Novara R, Pallara E, Cotugno S, et al. Onchocerciasis: Current knowledge and future goals. Front Trop Dis. 2022 Nov 17;3:986884.
6. Lakwo T, Oguttu D, Ukety T, Post R, Bakajika D. Onchocerciasis Elimination: Progress and Challenges. RRTM. 2020 Oct;Volume 11:81–95.
7. Diawara L, Traoré MO, Badji A, Bissan Y, Doumbia K, Goita SF, et al. Feasibility of Onchocerciasis Elimination with Ivermectin Treatment in Endemic Foci in Africa: First

Evidence from Studies in Mali and Senegal. Basáñez MG, editor. PLoS Negl Trop Dis. 2009 Jul 21;3(7):e497.

8. Tekle AH, Elhassan E, Isiyaku S, Amazigo UV, Bush S, Noma M, et al. Impact of long-term treatment of onchocerciasis with ivermectin in Kaduna State, Nigeria: first evidence of the potential for elimination in the operational area of the African Programme for Onchocerciasis Control. Parasites & Vectors. 2012 Feb 7;5(1):28.
9. Traore MO, Sarr MD, Badji A, Bissan Y, Diawara L, Doumbia K, et al. Proof-of-principle of onchocerciasis elimination with ivermectin treatment in endemic foci in Africa: final results of a study in Mali and Senegal. PLoS Negl Trop Dis. 2012;6(9):e1825.
10. Ojurongbe O, Akindele AA, Adeleke MA, Oyedeji MO, Adedokun SA, Ojo JF, et al. Co-endemicity of Loiasis and Onchocerciasis in Rain Forest Communities in Southwestern Nigeria. Ngondi JM, editor. PLoS Negl Trop Dis. 2015 Mar 26;9(3):e0003633.
11. Adeleke M, Sam-Wobo SO, Mafiana C, Olatunde GO. Perception on bioecology of onchocerciasis vectors around Osun River, South-western Nigeria. J Public Health Epidemiol. 2011;3(4):162–6.
12. Busari LO, Adeleke MA, Surakat OA, Akindele AA, Fasasi, Ojurongbe OO. Black Flies and Onchocerciasis: Knowledge, Attitude and Practices Among Inhabitants of Alabameta, Osun State, Southwestern, Nigeria. PLOS Negl. Trop. Dis; 2020.
13. Domche A, Nana-Djeunga HC, Yemeli LD, Nanga CL, Boussinesq M, Njiokou F, et al. Knowledge/perception and attitude/practices of populations of two first-line

communities of the Centre Region of Cameroon regarding onchocerciasis and black fly nuisance and bio-ecology. Parasites Vectors. 2021 Dec;14(1):546.

14. Eyo J, Onah I, Ubachukwu P, Ivoke N, Ekeh F. Effects of climatic conditions on the biting density and relative abundance of Simulium damnosum complex in a rural Nigerian farm settlement. Ann Agric Environ Med. 2014 Nov 26;21(4):697–700.

15. Otabil KB, Gyasi SF, Awuah E, Obeng-Ofori D, Tenkorang SB, Kessie JA, et al. Biting rates and relative abundance of Simulium flies under different climatic conditions in an onchocerciasis endemic community in Ghana. Parasites & Vectors. 2020 May 6;13(1):229.

16. Thomsen JS, Sonne M, Benfeldt E, Jensen SB, Serup J, MennE T. Experimental itch in sodium lauryl sulphate-inflamed and normal skin in humans: a randomized, double-blind, placebo-controlled study of histamine and other inducers of itch. Br J Dermatol. 2002 May;146(5):792–800.

17. Yirga D, Hajito K, Wondafrash M, Kassahun W, Deribe K. Knowledge and Belief about Cause and Prevention of Onchocerciasis in Bebeka, Southwest Ethiopia. Ethiop J Health Sci. 2008 Nov 1;18.

18. Adeoye AO, Ashaye AO, Onakpoya OH. Perception and Attitude of People Toward Onchocerciasis (River Blindness) in South Western Nigeria. Middle East Afr J Ophthalmol. 2010;17(4):310–4.

19. Surakat OA, Sam-Wobo SO, Ademolu KO, Adekunle MF, Adekunle ON, Monsuru AA, et al. Assessment of community knowledge and participation in onchocerciasis programme, challenges in ivermectin drug delivery, distribution and non-compliance

in Ogun State, southwest Nigeria. Infection, Disease & Health. 2018 Sep;23(3):137–45.

20. Afolabi O, Okaka C, Oke I, Oniya M. Knowledge, Attitude and Perception of Onchocerciasis and Ivermectin Treatment in Idogun Community, Ondo State, Nigeria. British Journal of Medicine and Medical Research. 2016 Jan 10;13:1–7.
21. WHO. Guidelines for stopping mass drug administration and verifying elimination of human onchocerciasis: criteria and procedures. Geneva: World Health Organization; 2016
22. Brieger WR, Okeibunor JC, Abiose AO, Wanji S, Elhassan E, Ndyomugyenyi R, et al. Compliance with eight years of annual ivermectin treatment of onchocerciasis in Cameroon and Nigeria. Parasites & Vectors. 2011 Jul 27;4(1):152.
23. Nwoke BE, Onwuliri CO, Ufomadu GO. Onchocerciasis in Plateau State; Nigeria: ecological background, local disease perception & treatment; and vector/parasite dynamics. J Hyg Epidemiol Microbiol Immunol. 1992;36(2):153–60.

Supporting Information

S1 File. Structured Questionnaire used for the survey of knowledge, practices and level of participation in mass administration of medicines at Erin-Ijesha and Ipole-Iloro communities in Southwest, Nigeria.

Click here to access/download
Supporting Information
S1 File.docx
