# Supplementary material for: Knowledge and attitude of populations on blackflies and onchocerciasis and participation in mass drug administration in first-line communities near Erin-Ijesha and Arinta waterfalls, Southwest Nigeria
Source: PLoS Negl Trop Dis. 2025 Aug 14;19(8):e0013411. doi: 10.1371/journal.pntd.0013411 (PMC12367129; doi:10.1371/journal.pntd.0013411)
Supplement: Attachment — Submitted filename: Rebuttal letter.docx [file pntd.0013411.s006.docx]

Department of Animal and Environmental Science,

College of Science, Engineering and Technology,

Osun State University,

P,M.B 4494,

Osogbo,

Osun State, Nigeria.

5^th^ March, 2025.

The Editorial Manager,

PLOS Neglected Tropical Diseases

San Francisco, USA.

Dear Sir,

**REVISED MANUSCRIPT ON KNOWLEDGE AND ATTITUDE OF POPULATIONS ON BLACKFLIES AND ONCHOCERCIASIS AND PARTICIPATION IN MASS DRUG ADMINISTRATION IN FIRST-LINE COMMUNITIES NEAR ERIN-IJESHA AND ARINTA WATERFALLS, SOUTHWEST NIGERIA.**

The authors sincerely appreciate the time and effort that you and the reviewers have invested in evaluating our manuscript for publication in PLOS NTD. Based on the constructive feedbacks on the manuscript titled “Knowledge and attitude of populations on blackflies and onchocerciasis and participation in Mass Drug Administration in first-line communities near Erin-Ijesha and Arinta waterfalls, Southwest Nigeria”, I write to inform you that the authors have carefully addressed all comments raised by reviewers. The corrections are highlighted below and have been highlighted in yellow in the marked-up copy of the manuscript.

**REVIEWER’S COMMENTS**

**REVIEWER #2**

**Comment 1:** The title should be rephrase. "Knowledge and attitude of ....... on blackflies..."

**Response 1**: Thank you for this observation. The title has been rephrased to “Knowledge and attitude of populations on blackflies and onchocerciasis and participation in Mass Drug Administration in first-line communities near Erin-Ijesha and Arinta waterfalls, Southwest Nigeria.

**Comment 2:** This statement is contradictory to the sentence on lines 40-42 that the region is previously unmapped for onchocerciasis transmission. Can authors clarify how MDA was initiated in an area that was not mapped?

**Response 2:** Thank you for this comment. According to information from the state NTD coordinators prior to the study, there is no documented information on blackfly infectivity and prevalence of onchocerciasis in study communities despite being at high risk of onchocerciasis due to proximity to breeding site. However, the “previously unmapped” has been removed and the sentence rephrased. Check lines 42-44.

**Line 42-44:** Preliminary assessments have identified blackfly biting activity at Erin-Ijesha and Arinta waterfalls in Southwest Nigeria, predisposing first-line communities to a high risk of onchocerciasis.

**Comment 3:** Abbreviate the genus after first usage and in middle of sentence

**Response 3:** This has been addressed in line 75.

**Comment 5**: This is not "current". Nigeria's treatment number is higher. please revise sentence.

**Response 5:** Thanks for this observation. This has been revised based on WHO 2024 data. Check lines 82-83.

**Line 82-83:** Currently, ivermectin has been administered to over 45 million Nigerians achieving 80.6% geographical coverage (WHO, 2024).

**Comment 4:** Given that individual communities are not mapped for onchocerciasis epidemiology, and impractical to do and that Nigeria has adopted the state-level an implementing unit for onchocerciasis, authors should provide the onchocerciasis endemicity information of the LGAs these communities belong to. This will provide better context to readers than the village data.

**Response 4:** Thanks for raising this observation. Again, the phrase previously unmapped was used based on lack of information on endemicity level at these LGAs according to the state coordinators. However, authors agree with reviewer and this has been removed and sentences properly revised. Check lines 105-113.

**Lines 105-113**: This study was conducted between October and November 2024 in two frontline communities near the Arinta and Olumirin waterfalls. The first site, Erin Ijesha (N7.569368, E4.891340), located in Oriade Local Government Area, Osun State, is the nearest community to the Erin-Ijesha waterfall. The second site, Ipole Iloro (N7.586372, E4.936383), situated in Ekiti West Local Government Area, Ekiti State, is the frontline community to the Arinta waterfall. There is no documented information on the level of onchocerciasis endemicity in the LGAs where these communities are located. However, blackfly activity observed during a preliminary assessment of the waterfalls close to the communities prompted the selection of these locations for the study.

**Comment 5:** What was the method of selection? Random, systematic, or purposively?

**Response 5:** The study communities were purposively selected as they were first-line communities to a breeding site. This has been added to the sentence. Lines 137-138.

**Line 137-138**: This community-based cross-sectional study was conducted in two purposively selected first-line communities between October 2024 and November 2024.

**Comment 6:** Authors should provide the correct English text of these responses

**Response 6**: Thanks for raising this. This has been done.

**Comment 7:** The number of rounds of MDA is not necessarily the same as dose. And given the leading question was about rounds, authors should maintain consistency. Furthermore, it would have been great to understand how consistent was the participation? Were those that participated in 5 rounds, consecutive five rounds, or total number of participation**.**

**Response 7:** Thanks for this comment. Since leading questions was about rounds and not dose, authors have revised appropriately. Also, it is total rounds received and this is reflected now. Check line 206-212.

**Line 206-212:** Among those aware, 113 (75.3%) in Erin-Ijesha and 66 (77.6%) in Ipole-Iloro reported receiving at least one round of ivermectin. When asked about the total number of ivermectin rounds received, 23 (15.3%) of respondents in Erin-Ijesha reported receiving one round, 31 (20.7%) two rounds, 30 (20.0%) more than two rounds, and 29 (19.3%) more than five rounds. At Ipole-Iloro, 16 (24.2%) had received one round, 11 (16.7%) two rounds, 22 (33.3%) more than two rounds, and 17 (25.8%) more than five rounds.

**Comment 8:** rephrase "communities at risk of transmission"

Is this just communities at risk or actual endemic communities?

**Response 8:** Thanks for this observation. It is actually endemic communities. This has been revised. Line 251.

**Comment 9:** Line 242: This should be moved to results section**.**

**Response 9:** Moved back to result section.

**Comment 10:** What sort of health risks and source? It would be beneficial to readers if authors can expatiate on this.

**Response 10**: The health risks and source has been added. Check line 276-279

**Line 271-274**: While effective, some of these substances pose health risks when applied to the skin or inhaled during application, potentially causing severe headaches, respiratory difficulties, skin irritation, or burns (Domche *et al*., 2021).

**Comment 11:** Authors should provided complete context of the insufficient community sensitization and education efforts alluded to here. Who is doing or responsible for this activity? Is such activity targeted at vector control in place? Providing more context will help readers with better appreciation of the information.

**Response 11**: Thanks for this comment. The community drug distributors (CDDs) who are charged with community sensitization are been alluded to here and this has been added to the sentence to avoid ambiguity. Check line 293-295

**Line 287-289**: This limited knowledge of the vector is a reflection of the insufficient community sensitization and education efforts by the community drug distributors (CDDs).

**Comment 12**: As commented earlier, this reviewer expresses reservation to this conclusion. The options provided as symptoms of onchocerciasis are late stage disease symptoms that are more common in settings with high communal microfilarial load. And given the sustained mass treatment in the study area, it is not surprising these symptoms are not common and knowledge is low among participants. Ocular dermatitis as a symptom of onchocerciasis cannot be determine or recognize by average participants as it requires specialize skill (slip lamp or OCT technology). In fact, authors should report this as a limitation of the current study rather than presenting this as a significant shortfall to program.

**Response 12**: The authors believe that IEC materials for sensitization and awareness campaigns typically highlight the common manifestations of late-stage onchocerciasis. Considering that these are high-risk communities and have been participating in ivermectin MDA for years, participants were expected to be aware of some of these late-stage symptoms. This rationale informed the question and the inclusion of a follow-up question asking participants if they were aware that untreated infection could lead to blindness. In high-risk communities where ivermectin distribution has been ongoing for years, it is crucial for residents to understand the potential consequences of the disease if left untreated. The authors suggest that this awareness could help improve ivermectin acceptability and uptake.

**Comment 13**: Please rephrase

**Response 13:** Rephrased. Check line 295-298

Line 295-298: The lack of knowledge of these late-stage manifestations could potentially impact drug acceptability and uptake, thereby further increasing transmission risk. These findings further indicate shortfall in community health education and sensitization by CDDs during mass drug administration campaigns.

**Comment 14:** This reviewer does not agree with the authors on the usage of therapeutic coverage here. Therapeutic coverage in the context of MDA is limited to the number of individuals in a population that were treated in a single round. It is misleading to readers to carry this response to "at least one round" as the coverage in these communities and compared it to the WHO threshold. Authors should revise and rephrase accordingly.

**Response 14:** The sentence has been rephrased and comparison to WHO threshold removed. Check line 299-302.

**Line 299-302:** A notable finding from this study is the high percentage of individuals in both communities who were aware of and had received ivermectin. Majority of participants were aware of ivermectin with 75.3% in Erin-Ijesha and 76.7% in Ipole-Iloro reporting they had received at least one round of treatment.

**Comment 15:** This is a repetition of the results. Authors should delete

**Response 15:** Deleted

**Comment 16**: Authors did not present results to support this assertion

**Response 16:** The assertion was based on respondents who claimed they had not received ivermectin because they were either unaware of the distribution efforts or unavailable during the campaigns.

**Comment 17:** Again, authors need to rephrase this to prevent misinformation to readers. Illness and pregnancy are top exclusion criteria for ivermectin MDA. Drug distributors are actually not supposed/required to revisit for that round of MDA. It is a safety issue as advised by producer and global guidelines and not operational failure of distributors as being presented by authors.

**Response 17:** Thanks for this comment. The sentence has been rephrased. Check line 312-316

**Line 312-316:** These findings suggest that community drug distributors rarely follow up with individuals who miss treatment, limiting both treatment uptake and therapeutic coverage. Similar observations were reported by [20] where individuals excluded were not often revisited, hindering efforts to achieve inclusive coverage.

**Comment 18:** The results presented by authors show that awareness about ivermectin among participants is quite high. Given that ivermectin is not usually sold and only available through MDA in this kind of settings, can authors provide an explanation on the mismatch between knowledge about ivermectin and ''lack of sensitization" especially as ivermectin distribution through MDA is always preceded by community sensitization

**Response 18**: The possible explanation for the high uptake of ivermectin despite low knowledge of onchocerciasis could be the familiarity of community members with the CDDs, who are often known members of the community. This familiarity likely encourages ivermectin uptake, further emphasizing the effectiveness of engaging community members as CDDs. Additionally, many respondents claimed ivermectin also treats intestinal infections. This aligns with findings previously reported in Ethiopia. This has now been incorporated. Check line 304-308.

**Line 302-307**: A possible explanation for the high uptake of ivermectin despite low knowledge of onchocerciasis could be due to the familiarity of community members with the CDDs, who are often known members of the community. This familiarity likely encourages ivermectin uptake, further emphasizing the effectiveness of engaging community members as CDDs. Additionally, many respondents claimed ivermectin also treats intestinal infections. This aligns with findings previously reported in Ethiopia [4].

**REVIEWER #3 COMMENTS**

**Comment 1:** While these specific communities were previously unmapped, the authors should clarify whether the implementation unit, of which these communities are a part, had been mapped. It appears so. Can the authors comment on why Erin-Ijesha and Arinta were not mapped, considering the presence of waterfall and fast flowing waters in those areas.

**Response 1:** We appreciate your comment. As previously mentioned, ivermectin distribution is implemented across all Local Government Areas (LGAs) in the state. However, according to the state NTD coordinators prior to this study, there is no recorded data on blackfly infectivity or the prevalence of onchocerciasis in the study communities, despite their high risk due to proximity to the waterfalls. To prevent ambiguity, the phrase “previously unmapped” has been removed.

**Comment 2: Line 94:** The authors state that this study was part of a broader survey. It would be beneficial if the 'broader survey' is referenced, whether through a published paper, protocol, or other documentation that provides readers with context about the scope of this survey. If no such reference exists, consider including a brief description of the broader survey to give readers a clearer understanding.

**Response 2:** Broader survey because a separate entomological and parasitological assessment studies are ongoing in study communities to provide information on status and transmission risk of onchocerciasis. However, the word broader has been removed and sentence rephrased to avoid misunderstanding since results of these other studies are still ongoing. Check line 95-98

**Line 95-98**: This study was designed as an epidemiological survey to evaluate community knowledge, attitudes, and determine level of participation in ivermectin mass drug administration (MDA) in order to assess the onchocerciasis transmission risk in the region.

**Comment 3**: Sample Size Calculation: The sample size calculation was double-checked and found to be accurate. However, it may be helpful to include the formula (Cochran’s formula) for readers who are not well-versed in sample size calculations.

**Response 3:** Thanks for this observation. The formula has been included. Check line 142-149.

**Line 142-149:** In the absence of prior data on prevalence and community knowledge of blackflies and onchocerciasis, Cochran’s formula; n = (Z^2 * P(1-P)) / E^2 was used to determine sample size. Where n= the required sample size, Z= the standard normal deviate, usually set at 1.96 for a 95% confidence level, P = the expected populations with knowledge of onchocerciasis in the population, which is usually assumed to be 0.5 (50%) in the absence of any previous record and E = the desired level of precision, which is usually set at 0.05 (5%) for most studies. The estimated sample size was 385.

**Comment 4**: Ivermectin Uptake vs. Knowledge: While ivermectin uptake was high (>70%), the community’s knowledge of onchocerciasis transmission was low. What did the community believe they were being administered ivermectin for? Do the authors have any insights into this disconnect? If so, it would be interesting to include this information in the manuscript.

**Response 4**: The possible explanation for the high uptake of ivermectin despite low knowledge of onchocerciasis could be the familiarity of community members with the CDDs, who are often known members of the community. This familiarity likely encourages ivermectin uptake, further emphasizing the effectiveness of engaging community members as CDDs. Additionally, many respondents claimed ivermectin also treats intestinal infections. This aligns with findings previously reported in Ethiopia. This has now been incorporated. Check line 302-307.

**Line 302-307**: A possible explanation for the high uptake of ivermectin despite low knowledge of onchocerciasis could be due to the familiarity of community members with the CDDs, who are often known members of the community. This familiarity likely encourages ivermectin uptake, further emphasizing the effectiveness of engaging community members as CDDs. Additionally, many respondents claimed ivermectin also treats intestinal infections. This aligns with findings previously reported in Ethiopia [4].

**Comment 5:** Systematic Non-Compliance, line 289-290: The study suggests the presence of systematic non-compliance. Did the authors identify any specific groups or subpopulations that declined ivermectin treatment? If so, this information could provide valuable insights.

**Response 5:** Thanks for this submission. Based on responses, the populations who simply refuse the drug for no reasons in the study communities had no formal education. This information has been added. Check line 308-311.

**Line 308-311**: Among those who had not participated in the treatment programs, many were persistent drug refusers, citing no specific reasons for rejecting the medication. Majority of the people who simply refuse the drug in both communities had no formal education, further highlighting the importance of education on treatment uptake.

**Comment 6**: The results are presented solely as percentages, with no additional statistical analyses included in the results section or within the tables. However, lines 309–311 suggest that additional statistical tests may have been conducted to assess significance levels. If this is the case, the authors should highlight what analysis was used to generate the significance level, with appropriate justification for methods used. Also, the tables should be updated to reflect these analyses. Presenting only numbers and percentages may not be sufficiently robust for readers to fully interpret the findings and it's implications.

**Response 6**: The statistical tests done were stated in the data analysis. Chi-sqaure and t-tests were done to determine relationship between variable. Check line 159-161

**Line 159-161**: Descriptive statistics were performed, and relationships between variables were assessed using t-tests and chi-square tests, with proportions analyzed at a 95% confidence interval.

We appreciate the insightful comments and constructive feedback provided by the reviewers and editors. We believe that the revised version strengthens our study's contribution to the field and importance to the national onchocerciasis elimination program in Nigeria.

Thank you for your time and consideration. We hope that the reviewer would find the revised manuscript suitable for publication.

Yours faithfully,

Dauda Oluwadamilare Ganiu.
