# Supplementary material for: Knowledge and attitude of populations on blackflies and onchocerciasis and participation in mass drug administration in first-line communities near Erin-Ijesha and Arinta waterfalls, Southwest Nigeria
Source: PLoS Negl Trop Dis. 2025 Aug 14;19(8):e0013411. doi: 10.1371/journal.pntd.0013411 (PMC12367129; doi:10.1371/journal.pntd.0013411)
Supplement: Attachment — Submitted filename: Rebuttal_letter_auresp_2.docx [file pntd.0013411.s007.docx]

Department of Animal and Environmental Science,

College of Science, Engineering and Technology,

Osun State University,

P.M.B 4494,

Osogbo,

Osun State, Nigeria.

19^th^ June, 2025.

The Editorial Manager,

PLOS Neglected Tropical Diseases

San Francisco, USA.

Dear Sir,

**REVISED MANUSCRIPT ON KNOWLEDGE AND ATTITUDE OF POPULATIONS ON BLACKFLIES AND ONCHOCERCIASIS AND PARTICIPATION IN MASS DRUG ADMINISTRATION IN FIRST-LINE COMMUNITIES NEAR ERIN-IJESHA AND ARINTA WATERFALLS, SOUTHWEST NIGERIA.**

The authors sincerely appreciate the time and effort that have been invested in evaluating our manuscript for publication in PLOS NTD. Based on the constructive feedbacks on the above titled manuscript, I write to inform you that the authors have carefully addressed all comments raised by editors. Kindly find below detailed responses while changes based on the corrections have been highlighted in yellow in the marked-up copy of the manuscript.

**EDITOR’S COMMENTS**

**Comment 1:** The authors addressed most of the reviewer’s comments. However, the editor has still some concerns about this ms. Line 82/83 Reference 10 clearly states that the reported population treated in Nigeria in 2023 was 34.9 million. What do the authors mean with ‘Currently, ivermectin has been administered to over 45 million Nigerians…’? [10]

**Response 1**: Thank you for this observation. The authors acknowledge this error. The 45 million was inadvertently written not for the actual reported treated population which remains 34.9 million but rather for the total population requiring MDA. This has been corrected in the manuscript. Check line 82

Line 82: Currently, ivermectin has been administered to over 34 million Nigerians achieving 80.6% geographical coverage [10].

**Comment 2:** Table 1. What do the authors mean with ‘Numbers’? Number of study participants?

**Response 2:** Thanks for this observation. Yes, the ‘numbers’ intended is the number of study participants and this has been affected in the manuscript. Kindly refer to Table 1.

**Comment 3:** Table 3: Onchodermatitis and itching are the most common signs of onchocerciasis. Why was this not included? How is ‘ocular dermatitis’ defined? Do the authors mean periorbital dermatitis? Ocular disease caused by O. volvulus that can be easily seen is sclerosing ceratitis. How would this be included in the table. How is blindness defined?

**Response 3:** Thank you for this observation. Itching has been invariably included as part of the questions in Table 2 on the immediate effect of black fly bite. Additionally, ocular dermatitis and blindness were explained to participants in their native language simply as the visual discomfort characterized by the redness, itching and swelling of the eyes while blindness as loss of vision. Accordingly, the term “ocular dermatitis/blindness” in the manuscript has been revised to “blindness/loss of vision” for clarity.

**Comment 4:** Line 199. Why did the authors introduce the phrase mass administration of medicine (MAM)? What is the difference to the commonly used term mass drug administration (MDA)?

**Response 4:** Thanks for this observation. Some implementing partners now use MAM interchangeably with MDA. However, to align with internationally accepted phrase, MAM in the manuscript has now been changed to mass drug administration (MDA). Check line 199.

**Comment 5:** Table 4. CDTi is a special form of MDA. What is the difference between CDTi and MAM?

**Response 5:** Thank you for this great observation. What the authors implied and was asked was their participation in future MDA campaigns. CDTi has been changed to MDA.

**Comment 6:** Line 331. Why is knowledge of blackfly bioecology important to increase compliance to MDA? Many studies have shown that trust to community drug distributors is more important than disease specific knowledge. The authors may want to address this point more in detail in their discussion.

**Response 6:** Thanks for this question and observation. The knowledge of blackfly bioecology is paramount due to its public health implication. This is because lack of this knowledge is a risk factor for exposure to infection although it may not directly impact compliance to MDA. Given the community’s high-risk status due to proximity to breeding sites, assessing their understanding of blackfly bioecology is important in determining whether this knowledge gap may impact the adoption of protective measures.

**Comment 7:** In addition, the ms still contains typos (for example Table 2 blackflies vs black flies)

**Response 7:** Thanks for this observation. The authors have corrected these typos.

Once again, we appreciate the insightful comments and constructive feedback which we believe have greatly improved our manuscript. We believe that the revised version strengthens our study's contribution to the field and its importance to the national onchocerciasis elimination program in Nigeria.

Thank you for your time and consideration. We hope that the reviewer would find the revised manuscript suitable for publication.

Yours faithfully,

Dauda Oluwadamilare Ganiu.
