# Supplementary material for: Knowledge and attitude of populations on blackflies and onchocerciasis and participation in mass drug administration in first-line communities near Erin-Ijesha and Arinta waterfalls, Southwest Nigeria
Source: PLoS Negl Trop Dis. 2025 Aug 14;19(8):e0013411. doi: 10.1371/journal.pntd.0013411 (PMC12367129; doi:10.1371/journal.pntd.0013411)
Supplement: Attachment — Submitted filename: Reviewer comment - PLOS NTD - 01.28.2025.docx [file pntd.0013411.s003.docx]

**Knowledge and attitude on blackflies and onchocerciasis and participation in MDA in frontline communities near Erin-Ijesha and Arinta waterfalls, Southwest Nigeria**

This study reports that preliminary assessments conducted in previously unmapped locations for onchocerciasis transmission identified blackfly biting activity in two foci in southwest Nigeria. Consequently, the authors undertook this study to evaluate the community’s perception and knowledge of blackflies, as well as onchocerciasis transmission and treatment. The findings from this research could provide valuable insights into the community’s understanding of onchocerciasis transmission, which can be leveraged to enhance the uptake of ivermectin treatment in affected communities. Below are a few comments for consideration.

1. Line 94: The authors state that this study was part of a broader survey. It would be beneficial if the 'broader survey' is referenced, whether through a published paper, protocol, or other documentation that provides readers with context about the scope of this survey. If no such reference exists, consider including a brief description of the broader survey to give readers a clearer understanding.
2. Ivermectin Uptake vs. Knowledge: While ivermectin uptake was high (>70%), the community’s knowledge of onchocerciasis transmission was low. What did the community believe they were being administered ivermectin for? Do the authors have any insights into this disconnect? If so, it would be interesting to include this information in the manuscript.
3. Sample Size Calculation: The sample size calculation was double-checked and found to be accurate. However, it may be helpful to include the formula (Cochran’s formula) for readers who are not well-versed in sample size calculations.
4. Systematic Non-Compliance, line 289-290: The study suggests the presence of systematic non-compliance. Did the authors identify any specific groups or subpopulations that declined ivermectin treatment? If so, this information could provide valuable insights.
5. While these specific communities were previously unmapped, the authors should clarify whether the implementation unit, of which these communities are a part, had been mapped. It appears so. Can the authors comment on why Erin-Ijesha and Arinta were not mapped, considering the presence of waterfall and fast flowing waters in those areas.
6. The results are presented solely as percentages, with no additional statistical analyses included in the results section or within the tables. However, lines 309–311 suggest that additional statistical tests may have been conducted to assess significance levels. If this is the case, the authors should highlight what analysis was used to generate the significance level, with appropriate justification for methods used. Also, the tables should be updated to reflect these analyses. Presenting only numbers and percentages may not be sufficiently robust for readers to fully interpret the findings and it's implications.
